# Evaluation of the Use of an Inorganic Bone Matrix in the Repair of Bone Defects in Rats Submitted to Experimental Alcoholism

**DOI:** 10.3390/ma13030695

**Published:** 2020-02-04

**Authors:** Iris Jasmin Santos German, Karina Torres Pomini, Ana Carolina Cestari Bighetti, Jesus Carlos Andreo, Carlos Henrique Bertoni Reis, André Luis Shinohara, Geraldo Marco Rosa Júnior, Daniel de Bortoli Teixeira, Marcelie Priscila de Oliveira Rosso, Daniela Vieira Buchaim, Rogério Leone Buchaim

**Affiliations:** 1 Department of Biological Sciences (Anatomy), Bauru School of Dentistry, University of São Paulo (USP), Bauru, São Paulo 17012-901, Brazil; irish_knaan@hotmail.com (I.J.S.G.); karinatorrespomini@gmail.com (K.T.P.); anacarolinacb25@gmail.com (A.C.C.B.); jcandreo@usp.br (J.C.A.); andreshinohara@yahoo.com.br (A.L.S.); marcelierosso@usp.br (M.P.d.O.R.); 2 Department of Dentistry, Faculty of Health Science, Universidad Iberoamericana (UNIBE), Santo Domingo 10203, Dominican Republic; 3 Mother and Teacher Pontifical Catholic University (PUCMM), Santo Domingo 10203, Dominican Republic; 4 Postgraduate Program in Structural and Functional Interactions in Rehabilitation, University of Marilia (UNIMAR), Marília, São Paulo 17525-902, Brazil; carloshbreis@yahoo.com.br (C.H.B.R.); daniel.dbt@hotmail.com (D.d.B.T.); danibuchaim@usp.br (D.V.B.); 5 University of the Ninth of July (UNINOVE), Bauru, São Paulo 17011-102, Brazil; geraldomrjr@yahoo.com.br; 6 University of the Sacred Heart (USC), Bauru, São Paulo 17011-160, Brazil; 7 Medical School, University Center of Adamantina (UniFAI), Adamantina, São Paulo 17800-000, Brazil

**Keywords:** bone repair, biomaterial, alcoholism, alcohol

## Abstract

To assess the effects of chronic alcoholism on the repair of bone defects associated with xenograft. Forty male rats were distributed in: control group (CG, *n* = 20) and experimental group (EG, *n* = 20), which received 25% ethanol ad libitum after a period of adaptation. After 90 days of liquid diet, the rats were submitted to 5.0-mm bilateral craniotomy on the parietal bones, subdividing into groups: CCG (control group that received only water with liquid diet and the defect was filled with blood clot), BCG (control group that received only water with liquid diet and the defect was filled with biomaterial), CEG (alcoholic group that received only ethanol solution 25% *v*/*v* with liquid diet and the defect was filled with blood clot), and BEG (alcoholic group that received only ethanol solution 25% *v*/*v* with liquid diet and the defect was filled with biomaterial). In the analysis of body mass, the drunk animals presented the lowest averages in relation to non-drunk animals during the experimental period. Histomorphologically all groups presented bone formation restricted to the defect margins at 60 days, with bone islets adjacent to the BCG biomaterial particles. CEG showed significant difference compared to BEG only at 40 days (17.42 ± 2.78 vs. 9.59 ± 4.59, respectively). In the birefringence analysis, in early periods all groups showed red-orange birefringence turning greenish-yellow at the end of the experiment. The results provided that, regardless of clinical condition, i.e., alcoholic or non-alcoholic, in the final period of the experiment, the process of bone defect recomposition was similar with the use of xenograft or only clot.

## 1. Introduction

The concept of alcoholism peaked in the eighteenth century, shortly after the growing production and marketing of distilled alcohol, resulting from the industrial revolution. Ethanol is a water-soluble organic solvent with the ability to penetrate all compartments of the human body, and its systemic effects produce changes in the central nervous system, muscular system, liver disease, chronic pancreatitis, cardiovascular disease, lung disease, among others [1].

Ethanol impairs bone formation by inhibiting osteoblast proliferation. In addition, alcohol induces oxidative stress and participates in the regulation of osteoclast differentiation, resulting in increased signaling of RANKL-RANK, kB nuclear factor activating receptor ligand in bone cells, and increased osteoclastogenesis [2].

In addition, ethanol changes the levels of cytokines responsible for regulating bone metabolism, such as increased tumor necrosis factor alpha (TNF-alpha) and interleukin-6 (IL-6). These, in turn, cause the suppression of osteoblast synthesis and consequently the deposition of osteoid matrix [3].

Stimulation of ethanol on osteocytes causes increased secretion of sclerostin protein, which binds to cellular receptors in order to antagonize the action of BMP in the Wnt signaling cascade. The morphology and osteocyte apoptosis number are also altered with alcohol consumption, which sends signs of osteoclast recruitment, increasing bone resorption [4].

This change in bone remodeling may be expressed by bone loss and serum osteocalcin levels, a marker of bone formation. Moreover, it compromises collagen gene expression, also non-collagenous matrix proteins, and significantly reduces the levels of carboxy-terminal procollagen I propeptide [5]. These changes are also related to the duration and amount of alcohol exposure [6].

Alcohol consumption causes harmful effects on bone density [7] and is related to osteoporosis, due to imbalance in bone repair [8], altering bone microarchitecture, decreasing trabecular and cortical bone thickness, and consequently increasing the risk of fracture [9].

There are many scientific reports about xenografts in the bone repair process, whose results evidence that it is a viable alternative to autologous grafts [10]. There is currently a social and commercial incentive for alcohol consumption worldwide, which may lead to a larger number of patients using ethyl derivatives in clinical practice. Given this context, associated with the lack of experimental studies in the literature that relate alcoholism to biomaterial used in this innovative research and translational projection, it was decided to analyze a low-cost bone substitute used in the areas of dentistry and medicine with alcoholism.

Therefore, the aim of this study was to evaluate the effects of chronic alcoholism on the repair of bone defects in rats filled or not with a bovine bone matrix.

## 2. Materials and Methods

### 2.1. Biomaterial—Bone Graft Substitutes

The Bonefill biomaterial (Bionnovation Biomedical A.B., Bauru, São Paulo, Brazil) evaluated in this study is produced by decalcification of the cortical portion of bovine femur, totally denatured and sterilized by 25 kGy gamma radiation. It is commercially available in average particle size between 0.6–1.5 mm in diameter and 0.5 gr package. This dental product is registered in Brazilian Ministry of Health by The National Sanitary Surveillance Agency (ANVISA10392710012).

### 2.2. Experimental Design

Forty adult 60-day-old male Wistar rats (*Rattus norvegicus*) were used, weighing approximately 250 g. The animals were kept in conventional cages containing 4 animals/box. The macroenvironment presented artificial timer-managed lighting, which controlled the 12/12-h light/dark cycle, with 220 lux brightness, 55% humidity, exhaust fan and air conditioning, maintaining an average temperature of 22 °C. All animals had free access to standard rat chow (Nuvilab^TM^, Nuvital, Colombo, Paraná, Brazil) and their daily consumption was not measured (Table 1). All experimental procedures on the animals were conducted after approval by the Institutional Review Board on Animal Studies of Bauru School of Dentistry, University of São Paulo (Protocol: CEEPA-023/2012).

**Figure 1:**
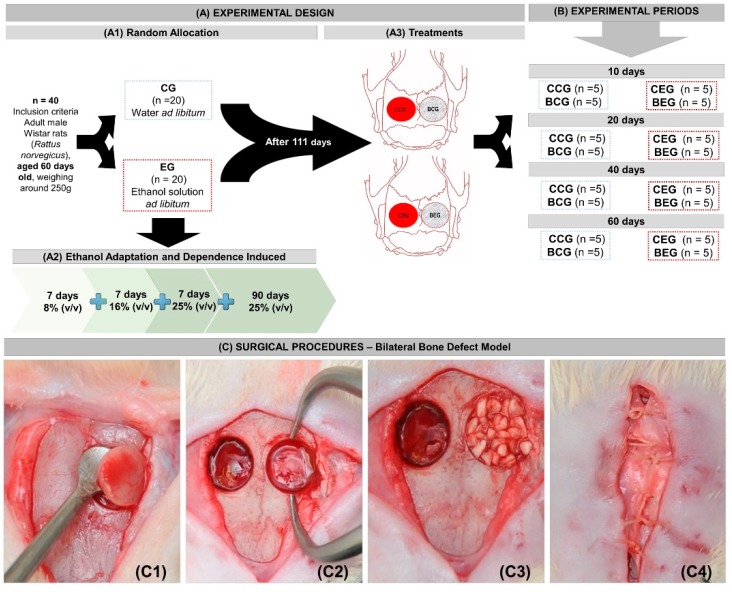
(**A**) Experimental design. (**A1**) Random Allocation—Forty adult male Wistar rats, (*Rattus norvegicus*), aged 60 days old, weighing around 250 g were divided into two broad groups: CG—Control group (*n* = 20)—that received only water with liquid diet and EG—Experimental group (*n* = 20)—that received ethanol solution 25% (*v*/*v*) with liquid diet after adaptation period. (**A2**) Ethanol Adaptation and Dependence Induced—Animals were gradually drunk at progressive concentrations of ethanol solution (8–16–25% *v*/*v*). After 21 days of alcohol adaptation, the animals remained at 25% (*v*/*v*) until the surgical procedure for 90 days. (**A3**) Treatments—After surgical procedures—bilateral bone defect model in the parietals, four subgroups were preformatted according to treatment (blood clot vs. biomaterial) and clinical conditions (alcoholic vs. non-alcoholic): Animals that received only water with liquid diet: CCG (right parietal bone defect was filled with blood clot) and BCG (left parietal bone defect was filled with biomaterial); Animals that received ethanol solution 25% (*v*/*v*): CEG (left parietal bone defect was filled with blood clot); BEG (right parietal bone defect was filled with biomaterial). B) Experimental Periods—at 10, 20, 40 and 60 days the skulls of 5 animals/group were collected, totalizing 5 defects/period of each subgroup CCG, BCG, CEG and BEG. (C) Surgical Procedures–Bilateral Bone Defect Model–(**C1**) 5-mm left parietal osteotomy; (**C2**) Two bone defects in parietal bones; (**C3**) One defect filled with biomaterial and the contralateral only with blood clot; (**C4**) Periosteum suture with nylon 5-0.

The animals were randomly separated into two large groups according to the type of liquid diet (drinking water or ethanol): CG, *n* = 20 (control group - non-alcoholic), which received a standard rat chow and water ad libitum; and EG, *n* = 20 (experimental group–alcoholic), which received a standard rat chow and ethanol ad libitum. After the surgical procedure, the groups were subdivided according to the type of treatment (clot or biomaterial) and the clinical condition (alcoholic or non-alcoholic), as follows: CCG (control group that received only water with liquid diet and the defect was filled with blood clot), BCG (control group that received only water with liquid diet and the defect was filled with biomaterial), CEG (alcoholic group that received only ethanol solution 25% *v*/*v* with liquid diet and the defect was filled with blood clot), and BEG (alcoholic group that received only ethanol solution 25% *v*/*v* with liquid diet and the defect was filled with biomaterial) (Figure 1A1).

### 2.3. Ethanol Adaptation and Induced Dependence—Semi-Voluntary Ethanol Administration—Alcohol-Liquid Diet

This study followed the determined chronic alcoholism model of “semi-voluntary”, where alcohol was the only liquid food available. Animals in the experimental group (EG) were submitted to an alcohol adaptation model, where the only available liquid source was ethanol ad libitum. In the first 7 days, the animals received 8% (*v*/*v*) ethanol solution, in the second week 16% (*v*/*v*) and third week 25% (*v*/*v*). After this period of gradual adaptation, the animals remained on the 25% (*v*/*v*) liquid ethanol diet for further 90 days, when they underwent experimental surgery and remained on the same diet until the corresponding euthanasia period. Animal health was monitored daily (Figure 1A2,B).

### 2.4. Surgical Procedures

After 111 days of ethanol intake and induced dependence, all rats were weighed and subjected to intramuscular general anesthesia with ketamine at a dose of 50 mg/kg i.m. (Dopalen™, Ceva, Paulinia, São Paulo, Brazil) plus xylazine at the dose of 10 mg/kg i.m. (Anasedan™, Ceva, Paulinia, São Paulo, Brazil), with strict monitoring of anesthesia mainly in alcoholic animals [11].

After frontoparietal trichotomy and antisepsis with 2% chlorhexidine, a cranio-caudal longitudinal incision of approximately 20 mm in length was made for tissue exposure and divulsion. Circular bilateral osteotomy was performed on the parietal bones with a 5 mm diameter trephine drill (Neodent, Curitiba, Paraná, Brazil) at low speed (1500 rpm) under constant saline irrigation (Figure 1C1,C2).

The defect on the left parietal was filled with 14 mg of biomaterial (previously established in a pilot study) and the right parietal was filled only with blood Clot (Figure 1A3,C3). The periosteum was repositioned and sutured with Vicryl^TM^ polyglactin suture (Ethicon J&J, São Paulo, Brazil) 5-0 and the integument with 4-0 silk suture (Ethicon^TM^, J&J, São Paulo, Brazil) (Figure 1C4).

After the surgical procedure, the animals were placed under incandescent light for complete anesthetic recovery and submitted to a single intramuscular injection of enrofloxacin 2.5 mg/kg (Flotril™; Schering-Plough SA, Rio de Janeiro, Brazil) and intramuscular injections of 0.06 mg/kg dipyrone (Analgex™; Agener União, São Paulo, Brazil) for 3 days.

### 2.5. Collection of Specimens and Histological Procedures

Five animals from each group were euthanized using the aforementioned anesthetic overdose at the respective periods of 10, 20, 40 and 60 days (Figure 1B). The specimens were removed and fixed in 10% buffered formalin for 48 h and then demineralized in EDTA, a solution containing 4.13% Titriplex™ III (Merck KGaA, Darmstadt, Germany) and 0.44% sodium hydroxide, for a period of approximately 40 days. Then, the specimens were subjected to standard histological procedures and included in Histosec ™ (Merck KGaA, Darmstadt, Germany). Histological sections were obtained with 5 µm thickness prioritizing the defect centers for hematoxylin and eosin, Masson’s trichrome and picrosirius-red staining.

### 2.6. Body Mass Analysis

Body mass was determined by simple weighing on a Bel Mark 3500 precision scale (BEL^TM^ Analytical Equipment Ltda, São Paulo, Brazil), with maximum capacity of 3500 g and minimum of 200 g with the aid of a Styrofoam box for animal containment. Measurements occurred on the day of the surgical procedure (initial mass) and on the corresponding days of euthanasia (10, 20, 40, and 60 days).

### 2.7. Histomorphological and Histomorphometric Evaluation

The histologic sections were analyzed in the Histology Laboratory of Bauru School of Dentistry, University of São Paulo (São Paulo, Brazil) by light microscopy (Olympus model BX50, Tokyo, Japan) at approximate magnifications of ×4, ×10, ×40 and ×100. To establish a standard criteria for judgment, there was a training session with an experienced pathologist.

For histomorphological description of the bone defect area, the central region (edge-to-edge measurement) was considered with the aid of free-scale image capture system (DP Controller software (3.2.1.276 version, Olympus, Tokyo, Japan) to analyze tissue formation granulation, inflammatory infiltrate, formation of primary bone tissue and bone maturation.

A virtual overall image of the defect (Masson’s trichrome, ×10) was generated to quantify the volume density (%) of newly formed bone tissue and biomaterial by AxioVision Rel. 4.8 Ink (Carl Zeiss MicroImaging GmbH, Jena, Germany). For determination of volumetric density (%), the equation Vvi = AAi = Ai/A × 100 was considered, considering Vvi (volume density), AAi (area density), Ai (area filled with newly formed bone tissue or particle of biomaterial), A (total area examined) (Figure S1) [12].

Images from picrosirius-red stained sections were captured using a higher resolution digital camera Leica DFC 310FX (Leica^TM^, Microsystems, Wetzlar, Germany) connected to a confocal laser microscope Leica DM IRBE and capture system LAS 4.0.0 (Leica^TM^, Microsystems, Heerbrugg, Switzerland). The quality of newly formed bone in the defects was evaluated by the orientation pattern and width of the collagen fibers detected by the birefringence of polarization colors ranging from red-orange (primary disorganized bone tissue) to green-yellow (organized bone-lamellar bone tissue).

### 2.8. Statistical Evaluation of Data

Data on body mass, volume density (%) of newly formed bone tissue and biomaterial particle were expressed as mean ± standard deviation of the mean (SEM). All tests were performed using Statistica 10.0 software (StatSoft Inc., Tulsa, OK, USA) and the significance level was set at *p* < 0.05. The independent “t” test was used to compare the initial body mass in alcoholic and non-alcoholic groups, and the paired “t” test was applied to compare the initial and final body mass within the same group. The percentage of bone formation and biomaterial in groups in different periods was assessed by one-way ANOVA variance test (time) for independent samples, and Tukey’s post hoc test, at a significance level of *p* < 0.05. To compare the percentage of bone formation in drunk vs. non-alcoholic animals (CCG vs. CEG and BCG vs. BEG) in the different periods, the t-test was applied for independent samples, and post hoc Tukey test, at a significance level of *p* < 0.05. To compare the percentage of bone formation in animals of the same group for different treatments (CCG vs. BCG and CEG vs. BEG), at different periods, the paired t-test and post hoc Tukey test were applied, at a significance level of *p* < 0.05.

## 3. Results

### 3.1. Effects of Ethanol Administration on Behavioral and Clinical Profiles of Rats

Regarding the general clinical profile, all animals presented good physical condition throughout the experiment, with no signs of morbidity and no mortality rate. There was no infection in either group, nor in the surgical area. However, some animals in the experimental group-alcohol group showed changes in their behavioral profile, especially regarding the parameters of agitation, aggressiveness and exploratory activity.

**Figure 2:**
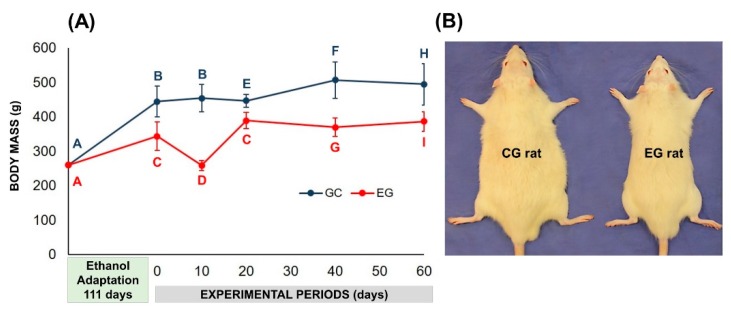
(**A**) Graphic representation of body mass (g) during ethanol adaptation-induced dependence (111 days) and experimental periods of control (CG-water ad libitum) and experimental (EG-ethanol solution ad libitum) groups. (**B**) Comparison between body masses of CG vs. EG showing the negative effect of alcohol. Different letters *p* < 0.05 (independent t-test and paired t-test showed interaction between group and period).

In the evaluation of body mass, after the period of alcoholic induction (111 days), the animals of the experimental group showed lower initial mass gain (day of surgery) compared to the control group (343.82 ± 41.93 vs. 444.59 ± 45.20, weight in grams, respectively). At 20 days, the mean mass of EG group showed no significant difference, but between 40–60 days there was a slight increase in the means from 5.9% to 6.4%, relative to day 0. In non-alcoholic animals, CG, the increase between 20–60 days was 7.9% to 14.1%, relative to day 0 (Figure 2).

### 3.2. Histological Evaluation

**Figure 3:**
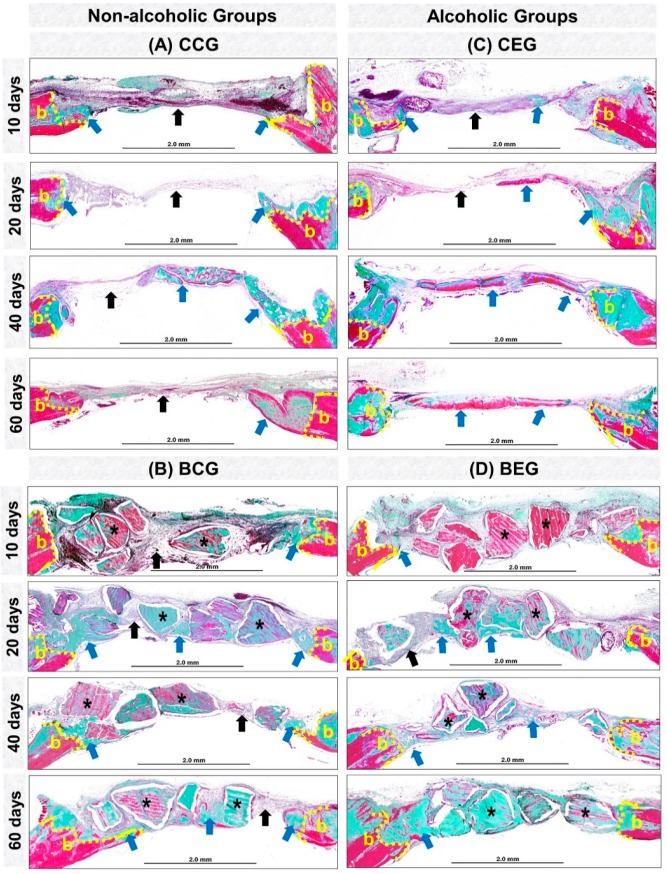
Panoramic histological views in skull defects created in the animals (**A–D**). Non-alcoholic (**A,B**) and alcoholic (**C**,**D**) treated with blood clot or biomaterial at different experimental periods, 10, 20, 40 and 60 days. (**A**,**B**) Non-alcoholic Groups: showed new bone formation (blue arrows) from the defect border (b), with partial closure by fibrous connective tissue in GCC (black arrows) or by particles of biomaterial surrounded by fibrous connective tissue in BCG (asterisk). (**C**,**D**) Alcoholic groups: new bone formation was observed from the border and on the dura-mater surface, with bone islets on the defect center in CEG and bone islets adjacent to the biomaterial particles in BEG. In both groups, at 60 days, lamellar tissue transition to compact tissue was observed (Masson’s Trichrome; original magnification ×4; bar = 2 mm).

**Figure 4:**
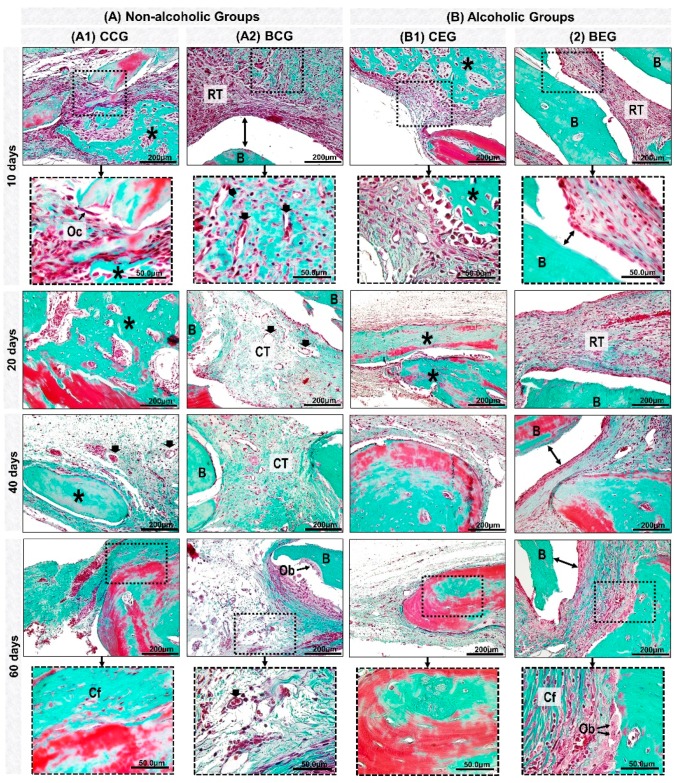
Details of evolution of bone healing of cranial defects created in the Non-Alcoholic (**A**) and Alcoholic (**B**) animals treated with blood clot or biomaterial. (**A1**) CCG (Non-alcoholic Group; defects filled with blood clot): at 10 days, defects showed trabecular bone formation (asterisk), with the presence of osteoclastic cells (Oc) on the edge of the remaining bone tissue. 20–40 days, the new bone formed showed bone maturation phase surrounded by blood capillaries (black arrow). At 60 days, collagen fibers were arranged in a more regular manner (Cf). (**A2**) BCG (Non-alcoholic Group: defects filled with biomaterial): at 10 days, defects showed tissue reaction (RT) surrounding the particles of the biomaterial (B); artifacts in histologic sections (double arrow–gap between biomaterial and tissue). Between 20–60 days, connective tissue (CT) presented scarce inflammatory cells with thin and thick collagen fibers, which were parallelly arranged at the end of the experimental period. (**B1**) CEG (Alcoholic Group; defects filled with blood clot): in the early periods, the defects presented inflammatory cells, decreasing at 40 days. The bone tissue formed at 60 days was predominantly compact and mature. (**B2**) BEG (Alcoholic Group; defects filled with biomaterial): 10–20 days, sections showed discrete bone formation, and biomaterial particles permeated by reaction tissue. In the later periods, collagen fibers were organized in parallel, and osteoblastic cells (Ob) forming a single cell line adjacent to the matrix. Masson’s Trichrome; original magnification ×40; bar = 100 μm; and Insets, magnified images ×100; bar = 50 μm.

**Figure 5:**
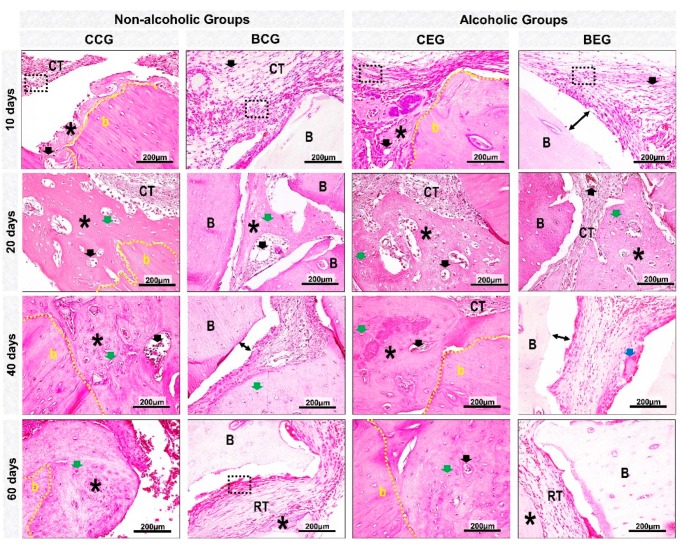
Histological details with HE staining of evolution of cranial defect bone healing created in the Non-Alcoholic and Alcoholic animals treated with blood clot or biomaterial. At 10-20 days, CCG and CEG showed the presence of richly vascularized connective tissue (CT) (black arrow) and new bone tissue (asterisk) at the defect margin (b) with trabecular arrangement. All groups presented inflammatory cells (inside the black lined area), more evident in BCG and BEG, permeating the particles (B). At 40 days there was typical lamellar arrangement, interspersed with osteocytes (green arrow), and in BCG and BEG inflammatory cells and multinucleated giant cells (blue arrow). At 60 days, bone tissue was mature and compact in defects filled with blood clot, there was decreased inflammatory reaction in BCG and BEG groups and regularly organized collagen fibers. Artifacts in histologic sections (double arrow–gap between biomaterial and tissue). HE; original magnification x 40; bar = 100 μm.

At 10 days, all experimental groups presented bone formation at the defect margins (Figure 3A–D). In groups treated with blood clot, CCG and CEG, there was predominance of richly vascularized granulation tissue filling the entire surgical area. However, BCG and BEG were shown to be reaction tissue surrounding the biomaterial particles (Figure 4A,B and Figure 5).

At 20 days, in the CCG and CEG groups, immature trabecular bone formation was in the transition phase of bone maturation, obtaining a denser lamellar arrangement at 40 days. In BCG, the reaction tissue was in resolution phase, with sparse inflammatory infiltrate, unlike that observed in the alcohol-treated / biomaterial-treated animals, BEG (Figure 4A,B and Figure 5).

At the end of the experimental period, at 60 days, all groups presented complete closure of the surgical area by fibrous connective tissue and / or particles of biomaterial. In defects treated with blood clot, in CCG and CEG, the new bone formation remained restricted to the defect margins and over the dura mater with a more evident bone maturation pattern, but with smaller thickness than the remaining bone. In the BCG and BEG groups, the particles were encased in evenly arranged, thicker collagen fibers (Figure 3A–D, Figure 4A,B and Figure 5).

### 3.3. Histomorphometric Evaluation

In relation to biomaterial volume density (%) in the BCG and BEG groups, a tendency of decrease between 10 (mean of 30.4%) to 60 days (mean of 18.98%) was observed (compare data in the Table 2). However, no statistical differences between periods in each group (ANOVA, *p* > 0.2) or groups per period (“t” test, *p* > 0.6) were observed.

Regarding bone formation volume density (Table 3), a significant increase was observed only in the defects created in alcoholic rats filled with blood clot, CEG group (*p* = 0.007), between 10 (mean of 6.67%) and 40 days (mean of 17.42%), as well as between 10 and 60 days (mean of 18.29%). In the same animals, the contralateral defects filled with biomaterials, BEG group, the bone formation volume density was 0.43 times smaller than CEG. Regardless of clinical condition (Table 4), no statistical differences in the bone formation were observed between CCG vs. CEG (*p* > 0.1) and BCG vs. BEG (*p* > 0.3).

### 3.4. Influence of Clinical Condition (Alcoholic Versus non-Alcoholic) and/or Type of Treatment (Clot vs. Biomaterial) on Collagen Content During the Bone Repair Process

**Figure 6:**
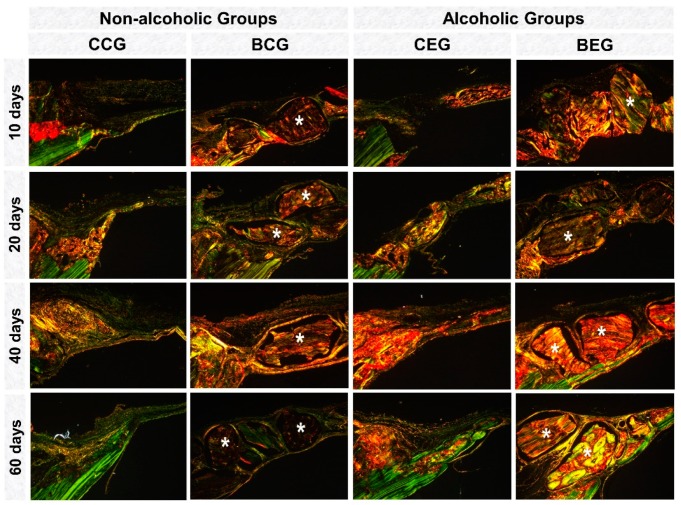
Photomicrographies of birefringent fibers stained with Picrosirius red under polarized light at 10, 20, 40 and 60 days of repair. In the initial periods, non-alcoholic groups, CCG and BCG showed red-orange collagen fibers becoming yellow-green at the end of the experiment. Alcoholic groups demonstrated evidently disorganized bone collagenous matrix, with red-orange birefringence around the grafted biomaterial (asterisk) and adjacent to the defect edges, CCG; BCG, at 60 days. The xenogeneic graft (BCG; BEG) presented red-orange birefringence. Picrosirius red staining, original magnification ×5. Scale bar = 50 μm.

Between 10–20 days, in the analysis of collagen matrix birefringence in polarized microscopy, all groups showed predominance of red-orange birefringence in the defect margins related to immature bone formation. In the BCG and BEG groups, the biomaterial presented red-orange birefringence surrounded by thin and disorganized collagen fibers (Figure 6).

In the final periods, 40–60 days, in non-alcoholic groups (CCG and BCG), the bone collagenous matrix was more organized with green-yellow birefringence than the alcoholic animals (Figure 6).

## 4. Discussion

The growing increase in chronic alcohol consumption in the last decades has encouraged the development of numerous researches in the medical and dental areas to alleviate the deleterious effects of ethanol on bone loss due to osteoporotic conditions or with difficulty in consolidating extensive bone defects [13,14].

However, there are still few studies in the scientific literature evaluating its impact on the repair and osseointegration process of biomaterials. Thus, the results of this in vivo study showed that the biomaterial served as a scaffold for bone cells, a biological event that can attenuate the harmful effects of ethanol on the bone repair process.

Experimentally several bone defect sites are tested, but in rats the most commonly used is the critical defect in calvaria. The experimental animal model used in this study involving 5-mm bilateral craniotomies in the parietal bones has been used in scientific research because of its ability to produce paired analyzes in a standardized manner and allow the evaluation of bone substitute materials in the reconstruction of critical size bone defects without involvement of the sagittal suture [15].

In addition, the bones of the calvaria and face have intramembranous ossification, a typical location of dental defects [16]. It is also possible to take into consideration the constitution of the biomaterial tested, being particulate and of medium size (0.6–1.5 mm), more commonly indicated in smaller defects because, in large orthopedic defects, typical of long bones, it is preferable to the use of block grafts.

Initial studies have graphically described a U-shaped curve relating ethanol consumption to various chronic diseases, but the literature still remains controversial regarding bone tissue effects [17]. Thus, we adopted in this experiment the 25% (*v*/*v*) alcohol dosage, as we based on preliminary analyzes by De Souza et al. [18], Buchaim et al. [19] and [20] who reported destructive effects on bone with the use of 20% and 25% (*v*/*v*) ethanol without inducing animal death, contrary to the 5% dosage that had a protective effect [21].

In addition, previous studies by our research group evaluated the plasma concentrations of three ethanol dosages and their effects on bone repair, which showed that 25% alcoholization had pharmacologically relevant plasma concentration (540 mg/dL) to cause alterations bones that compromised the morphofunctional restoration of lost tissue [19].

In this study, we evaluated the effects of alcohol on body mass and new bone formation by descriptive and histomorphometric analysis by Masson’s trichrome, and collagen fiber birefringence analysis by picrosirius-red staining in order to observe the alignment of the bone collagen and fiber structure at 10, 20, 40, and 60 days after injury.

The results of the body mass analysis showed lower averages in alcoholic than in non-alcoholic animals during the whole experimental period [20]. According to the literature consulted, the extensive use of alcohol can lead to dysfunctions in nutrient metabolism, causing changes such as decreased digestion and absorption, as alcohol has an influence on the stomach [22] and intestines [23] that can increase nutrient excretion, and consequently the risk of malnutrition [24,25]. Therefore, prolonged alcohol consumption may result in the lower body mass gained in animal experiments compared to non-alcoholic animals [26].

In the behavioral evaluation, some alcoholic rats initially presented aggressiveness and irritability, which are associated with hyperexcitability of the central nervous system, triggered by physiological dependence of alcohol [27,28].

All panoramic histological images of this experiment showed no integumentary invasion in the surgical bed, and the formation of fibrous connective tissue observed on the defect center and/or adjacent to the particles originated from the injured margins. Proper repositioning of the periosteum acted as a mechanical barrier preventing the collapse of extracranial tissues and consequently the migration of competing cells to osteoblasts [29,30].

Histomorphologically, defects filled with blood clot, CCG and CEG, presented the same pattern of bone repair, forming new bone tissue at the defect margins and extending centripetally, but with complete closure by fibrous connective tissue. This finding agrees with previous authors who suggest that it is a critical bone defect when spontaneous bone regeneration does not occur during the experimental period, requiring reconstruction of these defects by grafting [31].

In the initial periods, all experimental groups presented inflammatory infiltrate, a local defensive process against tissue aggression [32,33]. Between 20–40 days, the newly formed bone tissue was in transition from fine to compact trabeculae as observed by the study of Rocha et al. [34].

In the same period, BCG and BEG showed persistence of material implanted in the receptor bed with some dispersed inflammatory cells, without formation of macrophage aggregates and multinucleated giant cells, characteristic of chronic granuloma inflammatory process [35]. Thus, it is believed that the biomaterial proved to be biocompatible, i.e., the organism recognized the particles as part of its structure and not as an aggressor to its microenvironment [36,37].

At 60 days, the new bone formation remained restricted to the defect margins in all groups, with compact lamellar arrangement [38]. In BCG, bone islets adjacent to the particles were observed, which is consistent with studies that reported to be characteristic of osteoconductive biomaterial by providing a scaffold for osteoblastic cells, facilitating the deposition of new bone on its surface [39].

At the end of the experimental period, the inflammatory process present in the BCG and BEG groups was in the resolution phase, being more evident in non-alcoholic animals (BCG). These results lead to the perception of increase in serum levels of proinflammatory cytokines, derived from ethanol-induced liver disorders, which may have contributed to the persistence of the reaction tissue [40].

Histomorphometrically, in the analysis of the influence of time on bone formation, CEG showed a gradual increase in averages up to 40 and 60 days with statistically significant difference (Table 3, column). All other groups presented higher average in the final period of the experiment (60 days) in relation to the initial period (10 days), but without significant difference. Previous investigations have reported the physiological events that occur after the accommodation of biomaterial particles in the surgical bed, which alters the microenvironment and consequently delays the new bone formation [41].

Regarding the percentage of biomaterial in the BCG and BEG groups (Table 2, line), there was a decrease in final periods, but without significant difference. The delayed degradation of particles, as evidenced by their presence at 60 days, even in non-alcoholic animals, may be related to the intrinsic characteristics of the biomaterial [42]. This finding is corroborated by the study of Desterro et al. [43], who stated that non-sintered bovine apatites (<1000 °C) with organic matrix have lower dissolution rates, which directly impacts the biodegradation time.

Regarding the analysis of interference of the clinical condition, alcoholic and non-alcoholic, for each treatment, there was no statistical difference in all experimental groups (Table 4). In animals that used the biomaterial filling the bone defect, the results show that the formation of new bone was similar between the groups (BCG and BEG), regardless of the clinical condition (alcoholic or non-alcoholic), possibly for its osteoconductive property [39].

However, in comparing the percentage of bone formation according to treatment, clot or biomaterial (Table 3), CEG showed significant difference compared to BEG at 40 days (17.42 ± 2.78 vs. 9.59 ± 4.59, respectively). This fact may be correlated to the impact of ethanol on activities of the immune system, leading to changes in the phagocytic activity of polymorphonuclear cells. Thus, the persistence of particles may also have contributed to the delayed new formation in later periods compared to blood clot filling [44].

In the histochemical analysis of collagen fibers by picrosirius-red, all experimental groups initially presented red-orange birefringence, characteristic staining of formation of thin and disorganized collagen fibers turning greenish-yellow over the periods [45].

The biomaterial particles showed birefringence close to the newly formed bone tissue, precluding the measurement of specific fibers from bone repair. The study by Desterro [43] proved the presence of residual organic material in the particles of this biomaterial by X-ray diffraction analysis (XRD diffractogram of Bonefill^TM^), justifying the markup in this analysis.

Based on the experimental model employed, it can be concluded that, regardless of the clinical condition, alcoholic or non-alcoholic, in the final period of the experiment, the process of bone defect recomposition was similar with the use of xenograft or only clot. The use of biomaterial can provide a scaffold that guides bone growth, especially in larger defects.

For prospective research in the field of tissue engineering, it is suggested to associate with biomaterials, plasma-derived biodegradable polymers such as PRP, PRF and fibrin sealants in order to make graft material moldable in the surgical bed, facilitate insertion and agglutination, and prevent its dispersal [46]. In addition, adjuvant and noninvasive methods are also recommended to accelerate and improve the regeneration process such as hyperbaric chamber, pulsed ultrasound (LIPUS), and laser photobiomodulation therapy [46,47,48,49].

## 5. Limitations

Knowing that ethanol causes β-catenin signaling pathway dysregulation by increasing/decreasing the activity or expression of its protein constituents, suggests that future studies employ molecular and biochemical analyses in order to detail the effects of ethanol on filled bone defects with biomaterial.

## Figures and Tables

**Table 1:** Nutritional composition of the chow used in the experiment for all animals. Ad libitum feeding of chow.

Parameter	Chow *
Humidity (%)	2.30
Brute protein (%)	21.96
Ether (fatty) extract (%)	4.61
Mineral residue (%)	8.36
Brute fiber (%)	4.04
Nitrogen-free extract (%)	48.73
Calcium (%)	1.32
Phosphorus (%)	0.82
Brute energy (Kcal/kg)	3913

* Nuvilab CR1. Nuvital, Colombo, Paraná, Brazil.

**Table 2:** Mean ± standard deviation of volume density of comparison of biomaterial in the different experimental groups.

Group	10 Days	20 Days	40 Days	60 Days	One-Way ANOVA (p)
**BCG**	32.69 ± 11.71 ^a^	21.60 ± 5.16 ^a^	28.03 ± 10.64 ^a^	18.43 ± 11.86 ^a^	0.160
**BEG**	28.24 ± 11.60 ^a^	22.93 ± 11.46 ^a^	25.58 ± 4.00 ^a^	19.54 ± 11.06 ^a^	0.601
**Unpaired *t*-test** **(p)**	0.5875	0.8190	0.6426	0.8829	

Same lowercase letters indicate that there was no statistically significant difference. Significant differences *p* < 0.05.

**Table 3:** Mean ± standard deviation of volume density of new bone formation. Comparison among periods within the same group was evaluated by one-way ANOVA (column, 10 vs. 20 vs. 40 vs. 60 days). Comparison between defects treated with biomaterial vs. clot per condition (non-alcoholic and alcoholic) (line, CCG vs. BCG and CEG vs. BEG).

Period (Days)	Volume Density of New Bone Formation (%)					
	Non-Alcoholic Rat (n = 5/Period)			Alcoholic Rat (n = 5/Period)		
	CCG	BCG	Paired *t*-Test (*p*)	CEG	BEG	Paired *t*-Test (*p*)
10	5.30 ± 3.08 ^a^	7.54 ± 6.56 ^a^	0.326	6.67 ± 3,09 ^a^	6.98 ± 5,97 ^a^	0.852
20	8.41 ± 5.17 ^a^	13.79 ± 11.14 ^a^	0.370	12.33 ± 1,89 ^a,b^	7.96 ± 4,40 ^a^	0.156
40	15.50 ± 7.14 ^a^	12.97 ± 7.07 ^a^	0.610	17.42 ± 2,78 ^b^	9.59 ± 4,59 ^a^	0.018
60	14.51 ± 7.69 ^a^	13.34 ± 12.45 ^a^	0.865	18.29 ± 7,89 ^b,^*	12.85 ± 7,94 ^a,^*	0.122
One way ANOVA (p)	0.05	0.618		0.007	0.466	

Different letters (^a^ ≠ ^b^) indicate a statistically significant difference (*p* < 0.05), except for asterisks (*) that do not show significant difference (CEG vs. BEG; *p* = 0.122).

**Table 4:** Mean ± standard deviation of volume density of new bone formation Comparison between same treatment per animal condition (non-alcoholic and alcoholic). Unpaired t-test (line, CCG vs. CEG and BCG vs. BEG).

Period (Days)	Volume Density of New Bone Formation (%)					
	Clot			Biomaterial		
	CCG (n = 5/Period)	CEG (n = 5/Period)	Unpaired *t*-Test (*p*)	BCG (n = 5/Period)	BEG (*n* = 5/Period)	Unpaired *t*-Test (*p*)
10	5.30 ± 3.08 ^a^	6.67 ± 3.09 ^a^	0.528	7.54 ± 6.56 ^a^	6.98 ± 5.97 ^a^	0.899
20	8.41 ± 5.17 ^a^	12.33 ± 1.89 ^a^	0.151	13.79 ± 11.14 ^a^	7.96 ± 4.40 ^a^	0.307
40	15.50 ± 7.14 ^a^	17.42 ± 2.78 ^a^	0.591	12.97 ± 7.07 ^a^	9.59 ± 4.59 ^a^	0.678
60	14.51 ± 7.69 ^a^	18.29 ± 7.89 ^a^	0.464	13.34 ± 12.45 ^a^	12.85 ± 7.94 ^a^	0.942

Same lowercase letters indicate that there was no statistically significant difference. Significant differences *p* < 0.05.

## Supplementary Materials

The following are available online at https://www.mdpi.com/1996-1944/13/3/695/s1, Figure S1: Representative image of the methodology used to quantify the area of newly formed bone, graft particles, by Axio Vision software.
