# Supplementary material for: Evaluation of the Use of an Inorganic Bone Matrix in the Repair of Bone Defects in Rats Submitted to Experimental Alcoholism
Source: Materials (Basel). 2020 Feb 4;13(3):695. doi: 10.3390/ma13030695 (PMC7040897; doi:10.3390/ma13030695)
Supplement: Supplementary file 1 [file materials-13-00695-s001.pdf]

materials

MDPI

Supplementary

Evaluation of the Use of an Inorganic Bone Matrix in the Repair of Bone Defects in Rats Submitted to Experimental Alcoholism

Iris Jasmin Santos German [1,2,3], Karina Torres Pomini [1], Ana Carolina Cestari Bighetti [1], Jesus Carlos Andreo [1], Carlos Henrique Bertoni Reis [4], André Luis Shinohara [1], Geraldo Marco Rosa Júnior [5,6], Daniel de Bortoli Teixeira [4], Marcelie Priscila de Oliveira Rosso [1], Daniela Vieira Buchaim [4,7] and Rogério Leone Buchaim [1,4,*]

1. Department of Biological Sciences (Anatomy), Bauru School of Dentistry, University of São Paulo (USP), Bauru, São Paulo, 17012-901, Brazil; irish_knaan@hotmail.com (I.J.S.G.); karinatorrespomini@gmail.com (K.T.P.); anacarolinacb25@gmail.com (A.C.C.B.); jcandreo@usp.br (J.C.A.); andreshinohara@yahoo.com.br (A.L.S.); marcelierosso@usp.br (M.P.O.R.)
2. Department of Dentistry, Faculty of Health Science, Universidad Iberoamericana (UNIBE), Santo Domingo 10203, Dominican Republic
3. Mother and Teacher Pontifical Catholic University (PUCMM), Santo Domingo, Dominican Republic;
4. Postgraduate Program in Structural and Functional Interactions in Rehabilitation, University of Marilia (UNIMAR), Marília, São Paulo, 17525-902, Brazil; carloshbreis@yahoo.com.br (C.H.B.R.); daniel.dbt@hotmail.com (D.B.T.); danibuchaim@usp.br (D.V.B.)
5. University of the Ninth of July (UNINOVE), Bauru, São Paulo, 17011-102, Brazil; geraldomrjr@yahoo.com.br
6. University of the Sacred Heart (USC), Bauru, São Paulo, 17011-160, Brazil
7. Medical School, University Center of Adamantina (UniFAI), Adamantina, São Paulo, 17800-000, Brazil;

* Correspondence: rogerio@fob.usp.br

Received: 13 December 2019; Accepted: 27 January 2020; Published: 3 February 2020

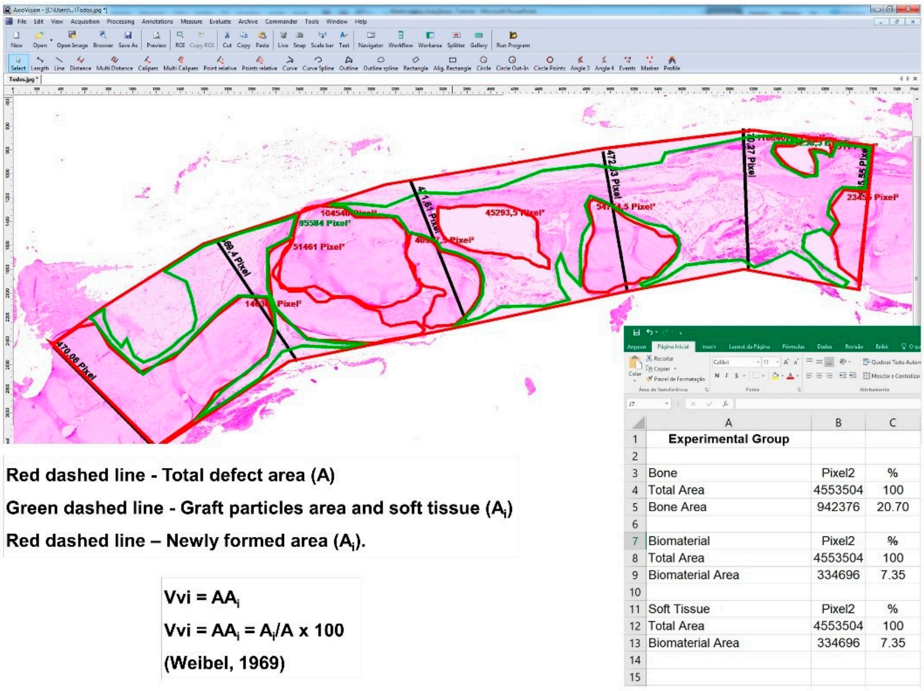

Figure S1: Representative image of the methodology used to quantify the area of newly formed bone, graft particles, by Axio Vision software.
